# Are white matter abnormalities associated with “unexplained dizziness”?

**DOI:** 10.1016/j.jns.2015.09.006

**Published:** 2015-11-15

**Authors:** Hena Ahmad, Niccolò Cerchiai, Michelangelo Mancuso, Augusto P. Casani, Adolfo M. Bronstein

**Affiliations:** a Academic Department of Neuro-otology, Division of Brain Sciences, Imperial College London, Charing Cross Hospital, London, United Kingdom; b Department of Medical and Surgical Pathology, Otorhinolaryngology Unit, Pisa University Hospital, Pisa, Italy; c Neurological Clinic, University of Pisa, Pisa, Italy

**Keywords:** Small vessel disease, White matter hyperintensities, Unexplained dizziness

## Abstract

**Introduction:**

Although cerebral small vessel disease is a significant contributor to the development of imbalance and falls in the elderly, whether it causes dizziness is not known.

**Methods:**

A retrospective case analysis was conducted for 122 dizzy patients referred to two neuro-otology tertiary centres in London and Pisa. Patients were divided into ‘explained’ causes of dizziness (e.g. benign positional vertigo, vestibular neuritis, orthostatic hypotension, cerebellar ataxias) and ‘unexplained’ dizziness. White matter hyperintensities (WMH) in MRI (T2 weighted and FLAIR sequences) were blindly rated according to the Fazekas scale.

**Results:**

122 patients; 58 (mean age = 72, SD = 7.95 years) in the ‘*unexplained*’ group and 64 (mean age = 72.01, SD = 8.28 years) in the ‘*explained*’ group were recruited. The overall frequency of lesions (Fazekas 1–3) significantly differed between groups (p = 0.011). The frequency of severe lesions (Fazekas 3) was significantly higher in the ‘*unexplained*’ group (22%) than in the ‘*explained*’ group (5%; p = 0.003).

**Conclusion:**

Increased severity of WMH in cases of unexplained dizziness suggests that such abnormalities are likely contributory to the development of dizziness. WM lesions may induce dizziness either because patients perceive a degree of objective unsteadiness or by a disconnection syndrome involving vestibular or locomotor areas of the brain.

## Highlights

• Retrospective analysis of 122 dizzy patients divided into explained and unexplained causes
• Severity of MRI white matter disease predicts unexplained dizziness, not vascular risk factors
• White matter lesions more severe in unexplained than in explained dizziness group
• White matter lesions likely contributory to development of *unexplained* dizziness

## 1 Introduction

Cerebral white matter disease (WMD) and its role in cognitive decline, falls and stroke [1], [2], has generated immense interest over the years. Although it is known that WMD is associated with gait and posture abnormalities, a link between white matter abnormalities and dizziness has not been established yet.

The diagnosis in older dizzy patients is challenging as there are often multifactorial causes [3] and a large proportion of patients remain undiagnosed. Patients often report non-specific dizziness, vague unsteadiness, disequilibrium or even light headedness. With WMD currently impacting 80% of the elderly population [4] and dizziness affecting approximately 30% of the population over 65 years of age [5], the significance of research in this area cannot be underestimated. A previous study by Day et al. [6] at the advent of routine MRI, recruited dizzy patients but did not find an appreciable difference between imaging of dizzy and non-dizzy patients. However, the numbers were small and the technique was new. Influential studies by Colledge et al. [7], [8] concluded there were no differences in imaging between dizzy and non-dizzy subjects although increased midbrain white matter (WM) lesions were noted. A very recent study in this journal, however, indicates that the presence of WM lesions is an independent predictor of residual dizziness in patients with previous vestibular neuritis [9]. In order to reassess whether WMD may directly contribute to dizziness, a retrospective cohort study was conducted.

## 2 Methods

Patients were included if they were aged between 45 and 90 years and were referred with dizziness to tertiary referral neuro-otology centres in London and Pisa. These are national centres of expertise in the diagnosis, investigation and management of dizziness and imbalance and are typically referred a combination of general and complex dizzy patients. “Dizzy” symptoms included true rotational vertigo, light-headedness, giddiness and a sense of non-specific unsteadiness. Patients with no dizziness, psychogenic or functional dizziness, no imaging available or CT scan only (n = 3) and age > 90 were excluded (n = 17). In total, 122 patients (59 males) aged 45–90 were recruited. Patients were referred from general practitioners, neurologists and ear nose and throat specialists from December 2010–2014. All patients (81 in London and 41 in Pisa) had been seen and examined by experienced neuro-otologists. For this study, the case notes were reviewed and patients were divided into two groups based on history, clinical examination findings and laboratory results. Group 1 consisted of patients with ‘*explained*’ causes of dizziness. These included benign positional paroxysmal vertigo (BPPV — history of brief, positional, rotational vertigo with positive Dix–Hallpike manoeuvre), vestibular neuritis (history of acute prolonged rotational vertigo, associated with nausea, vomiting and imbalance with spontaneous, horizontal, unidirectional, nystagmus, with torsional component, unilaterally positive head impulse test and no other neurological abnormality. Tests show unilateral reduction of nystagmic response on calorics i.e. > 25% canal paresis and normal audiometry). Also included were vestibular migraine (history of at least five episodes of vestibular symptoms (vertigo/head motion induced dizziness) with temporally associated migrainous headaches lasting from minutes up to 72 h), Meniere's disease (combination of vertigo, cochlear symptoms and low frequency sensorineural hearing loss), bilateral vestibular loss (bilateral absence of nystagmic response; < 10 °/s slow phase velocity on calorics/rotational chair testing) orthostatic hypotension (decrease in systolic blood pressure of 20 mm Hg or diastolic 10 mm Hg from lying to immediately on standing and after 3 min) stroke, cerebellar ataxias, Parkinson's disease, hydrocephalus and temporal bone fracture. Group 2 included patients with ‘*unexplained*’ dizziness, i.e. no recognised diagnosis which could account for the dizziness. For all patients both main and secondary diagnoses are listed in Table 1.

Type of dizziness and vascular risk factors (heart disease, hypertension, diabetes, hypercholesterolemia, obesity, smoking and previous stroke) were also noted.

Full neuro-otological examination included eye movements (spontaneous, gaze evoked and positional nystagmus, pursuit, saccades, Dolls head, head-impulse test), cerebellar and sensory examination. Specific attention was paid to the examination of gait and posture. Abnormalities of gait (eg. broad based/ataxic/apraxic) were recorded. Tandem gait was considered abnormal if patients were unable to perform the ‘heel to toe’ walk or if consistent stepping errors were present. Romberg's test was classified as negative, excessive sway or fall. Postural responses to push/pull of the upper trunk were rated as abnormal if subjects would fall if not supported or exhibited retropulsion/propulsion if corrective steps were absent or multiple steps were needed to maintain posture. All patients underwent laboratory tests including audiometry, bithermal calorics or rotation chair electronystagmography. All patients were also specifically asked about postural symptoms (dizziness caused by upright posture relieved by sitting or lying position). If present, postural blood pressure (supine, immediately on standing and after 3 min) was measured. Brain imaging (3 Tesla MRI) was reviewed by a neurologist blinded to the clinical details. White matter hyperintensities (WMH) on 122 MRI T2-weighted and fluid-attenuated inversion-recovery (FLAIR) sequences were rated according to the Fazekas scale [10]. This is a scale grading WMH burden according to severity (0: none or a single punctate lesion; 1: multiple punctate lesions; 2: early confluency of lesions; 3: large confluent lesions). (Of note, in all cases, the blind assessment was found to be in agreement with the independent neuro-radiologist report rating the white matter lesion load as mild, moderate or severe).

Primary outcome was the degree of WMH in the two groups. A Pearson's 2 × 3 chi-squared test was performed to analyse the difference in Fazekas scales between the groups. As a secondary analysis, the presence or absence of rotational vertigo and gait and posture abnormalities were analysed using a 2 × 2 chi-squared test. The degree of total (aggregate) vascular risk factors between groups was compared with a chi-squared test. Binary logistic regression was used to evaluate the significant predictors of unexplained dizziness. Statistical analysis was performed using SPSS 22 (SPSS, Inc.; Chicago, USA) with significance set at p < 0.05.

## 3 Results

**Fig. 1:**
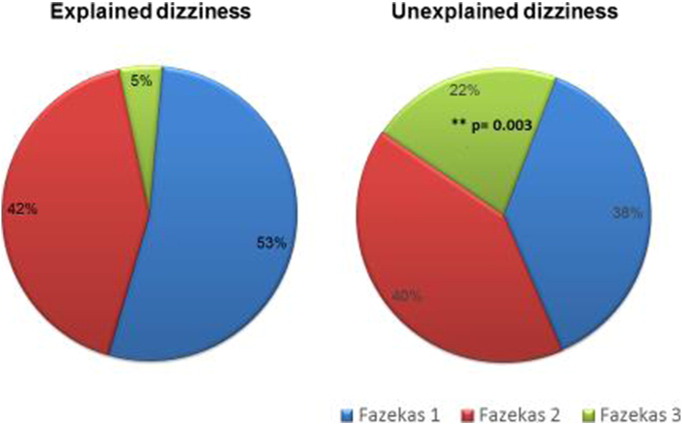
Severity of white matter disease on MRI (Fazekas scores), expressed as percentage of patients with “*explained*” and “*unexplained*” causes of dizziness.

There were 64 patients (mean age 72.01 years, SD = 8.28), in the “*explained*” group and 58 patients (mean 72 years, SD = 7.95) in the “*unexplained*” group. Fig. 1 summarises the Fazekas scores in the two groups. A significant difference was observed between the overall Fazekas scores in *explained* and *unexplained* dizzy groups (χ^2^ = 8.87, df = 2, p = 0.011). The frequency of severe lesions (Fazekas 3) was significantly greater in the unexplained group (22%) than in the explained group (5%); (χ^2^8.39, df = 1, p = 0.003).

Gait and posture abnormalities, (errors in tandem walk, gait dyspraxia, abnormal postural responses), were more frequent in the *unexplained* group (45%) as compared to the *explained* group (25%), (χ^2^ = 5.30, df = 1, p = 0.021). The presence of true rotational vertigo was significantly more frequent in the *explained* than in the *unexplained* group (χ^2^ = 9.60, df = 1, p = 0.003).

The total (aggregate) vascular risk factors were 65% in the *unexplained* group and 34% in the *explained* group (χ^**2**^ = 7.57, df = 1, p = 0.005). However, Fazekas score was found to be the *only* significant predictor of unexplained dizziness (binary logistic regression; p = 0.023). In particular, vascular risk factors were not found to be significant predictors of unexplained dizziness (p > 0.05).

## 4 Discussion

To our knowledge this is the first study to evaluate patients in specialist clinics to assess whether WMH is associated with unexplained dizziness. The relationship observed between increased severity (Fazekas 3) of WMH in our “*unexplained*” group suggests these abnormalities are likely contributory to the development of the dizziness. This is supported by the recent finding of WMH being a predictor of chronic dizziness in patients with previous vestibular neuritis [9].

It could be argued, however, that perhaps age or vascular risk factors were contributory to the unexplained dizziness. It should be noted though that our two groups were age-matched hence increased vascular risk factors cannot be solely attributed to age related changes. Also, despite increased frequency of ‘central’ pathology (eg. ataxias) observed in the ‘explained’ group the vascular risk factors were lower than in the unexplained group. Furthermore, our regression analysis indicates that the only predictor of unexplained dizziness was Fazekas score but not vascular risk factors. In support of this, a recent prospective cohort study [11] of community dwellers found that vascular risk factors combined could only explain 2% of variance in WMH suggesting a non-vascular aetiology for development of WMH, more so than previously considered.

It was also observed that the frequency of “true” rotational vertigo was significantly lower in the *unexplained* dizziness group with respect to the *explained* group, as expected from the frequent peripheral vestibular diagnoses in the latter group. However, recent studies suggest that the type (quality) of dizziness may contribute little to diagnosis [12], [13]. These features, however, will have to be confirmed in future prospective studies. It is important to highlight that we do not advocate the diagnosis of dizziness likely due to WMD on the basis of imaging findings alone, given that the presence of common and treatable causes of dizziness in this age group is high. In our sample, 26% of patients in the ‘*explained*’ group had moderate–severe (Fazekas 2–3) WMH and yet the main cause of the dizziness was BPPV, easily treated with a simple and inexpensive re-positioning procedure such as Epley or Semont manoeuvres.

We understand the limitations of this retrospective analysis, one of which is the accuracy of original recorded data. Patients, however, were seen by experienced neuro-otologists who performed a structured assessment minimising variability in record keeping. We also recognise that future studies are necessary for a normal control group to be prospectively assessed but in our study the *explained* dizzy group acted as a valid retrospective control group. Furthermore, quantifying WMH would be a more objective way of evaluating lesion burden and, perhaps more importantly, localization. Whilst this may form the basis of future work in this area, the Fazekas scale remains a validated tool in grading WM lesions [14], [15].

A previous, well conducted, prospective study [8] showed no major MRI differences between dizzy and non-dizzy subjects. It is, however, important to consider that firstly their subjects were recruited from the community and were not necessarily patients. Secondly, the imaging techniques were older, from the early 90's, and the association of WMH with objective balance dysfunction was not established then as it is now [16]. Our patients were recruited from tertiary neuro-otology clinics and are likely to have more relevant symptoms of dizziness hence explaining why structural abnormalities may not have been as prevalent in their study. The intensity of the dizziness, however, was not quantified with questionnaires in ours or previous studies and, again, this will have to be undertaken in future prospective studies.

## 5 Conclusion

We report that the severity of white matter small vessel disease is higher in elderly patients with no specific cause for their dizziness (“unexplained” dizziness). We postulate that white matter lesions may induce dizziness either because patients perceive a degree of objective unsteadiness or by a cortical–subcortical disconnection syndrome, secondary to disruption of white matter tracts involved in gait and balance control [16], [17]. Contrary to previous studies, our findings suggest that elderly patients with dizziness, without a clear diagnosis and despite clinical and vestibular assessment, should undergo brain imaging to assess the level of WMD. Currently treatment involves preventive measures including control of vascular co-morbidities [18]. Customised retraining through a combination of rehabilitation and possibly neuro-modulation of cortical pathways may form part of future potential treatment options for this subgroup of dizzy patients [19].

## Contributions

Both HA and NC contributed equally to the design, recruitment of patients, drafting and revising the manuscript. NC reviewed the case notes in Italy and London. MM reviewed the scans in Italy and HA reviewed the scans in London. AB conceived and designed the study, reviewed the manuscript and provided supervision.

## Funding

This study was supported by the Medical Research Council (MR/J004685/1), UK, the Imperial College London Biomedical Research Centre and the Mario Marinelli Foundation, Pisa, Italy.

## Competing interests

None.

## Ethics

This study was approved by the local research ethics committee, Imperial College London and Department of Medical and Surgical Pathology, Otorhinolaryngology Unit, Pisa University Hospital, Pisa, Italy.

## Figures and Tables

**Table 1:** Demographics and diagnoses of *unexplained* and *explained* dizziness groups. Values represent numbers of patients with percentage in parentheses.

Demographics	Group 1 N (%)	Group 2 N (%)
Unexplained 58 (48)	Explained 64 (52)
Age (years)	Range: 55–90 (Mean: 72, SD = 7.95)	Range: 45–90 (Mean: 72, SD = 8.28)
Male	28 (48)	31 (48)
Female	30 (52)	33 (51)

*Diagnosis*^a^		
BPPV	–	20 (31)
Vestibular neuritis	–	17 (26)
Vestibular migraine	–	10 (15)
Orthostatic hypotension	–	6 (9)
Cerebellar ataxia	–	6 (9)
Meniere’s disease	–	5 (7)
Bilateral vestibular failure	–	4 (6)
Stroke	–	2 (3)

*Other*		
Extrapyramidal syndrome, hydrocephalus, temporal fracture	–	3 (4)

*Past history/secondary diagnoses*		
Migraine	7 (11)	2 (3)
BPPV	5 (8)	5 (7)
Orthostatic hypotension	2 (3)	2 (3)

a More diagnoses than patients are listed in the explained group due to a proportion of patients with multiple diagnoses.
